# Stakeholder perspectives on factors influencing acute care patient outcomes: A qualitative approach to model refinement

**DOI:** 10.21203/rs.3.rs-3817903/v1

**Published:** 2023-12-29

**Authors:** Jessica Ziemek, Natalie Hoge, Kyla F Woodward, Emily Doerfler, Alison Bradywood, Alix Pletcher, Abraham D Flaxman, Sarah Iribarren

**Affiliations:** University of Washington School of Nursing; University of Washington School of Nursing; University of Washington School of Nursing; University of Washington School of Nursing; Washington State Board of Nursing; University of Washington Institute for Health Metrics and Evaluation; University of Washington Institute for Health Metrics and Evaluation; University of Washington School of Nursing

**Keywords:** Staffing, workforce, RN staffing, care models

## Abstract

**Background:**

Health systems have long been interested in the best practices for staffing in the acute care setting. Studies on staffing often focus on registered nurses and nurse-to-patient staffing ratios. There are fewer studies on the relationship between interprofessional team members or contextual factors such as hospital and community characteristics and patient outcomes. This qualitative study aimed to refine a causal model by soliciting hospital stakeholder feedback on staffing and patient outcomes.

**Methods:**

We conducted a qualitative study using semi-structured interviews and thematic analysis to understand hospital stakeholder perspectives and their experiences of factors that affect acute care inpatient outcomes. Interviews were conducted in 2022 with 38 hospital stakeholders representing 19 hospitals across Washington State.

**Results:**

Findings support a model of characteristics impacting patient outcomes to include the complex and interconnected relationships between community, hospital, patient, and staffing characteristics. Within the model, patient characteristics are nested into hospital characteristics, and in turn these were nested within community characteristics to highlight the importance of setting and context when evaluating outcomes. Together, these factors influenced both staff characteristics and patient outcomes, while these two categories also share a direct relationship.

**Conclusion:**

Findings can be applied to hospitals and health systems across the globe to examine how external factors such as community resource availability impact care delivery. Future research should expand on this work with specific attention to how staffing changes and interprofessional team composition can improve patient outcomes.

## INTRODUCTION

Health systems face ongoing challenges in recruiting and retaining staff to meet the needs of their patients. Best practices in acute care staffing have long been a topic of interest for organizations around the world, with more recent attention in the United States by legislative entities focused on ensuring the availability of a healthcare workforce that can meet the needs of their communities. Frequently, staffing studies focus exclusively on registered nurses (RNs) and show that a higher nurse-to-patient ratio is associated with improved patient outcomes.^1^ However, few studies have addressed the relationship between staffing of other interprofessional team members and patient outcomes, and even fewer have addressed contextual factors in organizations and communities that may influence staffing, patient outcomes, or both. A better understanding of the relationships between these factors is needed to ensure that policies and practices support optimal patient outcomes and to provide healthcare work environments conducive to quality care.

In 2021, the Washington state (WA) legislature passed a bill focused on transparency in healthcare.^2^ The bill directed the state Department of Health to commission an interdisciplinary team to examine the relationships between the acute care workforce and patient outcomes by systematically investigating how the number, type, education, training, and experience of staff affects patient mortality and other patient outcomes, utilizing scientifically sound research methods and input from stakeholders throughout the state.^2^ Our team, led by the University of WA School of Nursing in collaboration with researchers at the Institute for Health Metrics, was selected to conduct this study. To conduct this research, we established partnerships with hospital leaders, healthcare associations, and union representatives. The process included: (1) reviewing studies assessing the impact of workforce characteristics on patient outcomes, (2) developing a preliminary model and analysis plan based on the review, key informant input, and available data sources, (3) refining the model using stakeholder feedback, and (4) completing a quantitative analysis utilizing anonymized state- and hospital-collected healthcare data guided by the refined model.

The scoping review we completed in phase 1 identified that outside of RNs, healthcare team staffing is seldom quantified in health services literature, and contextual factors at the hospital and community level are rarely included when examining patient outcomes.^3^ The subsequent phases of our study aimed to understand what those missing factors were and how they played out in various acute care contexts, including impacts on operations, work environment, quality, and outcomes for patients and workers. The purpose of this paper is to present the perspectives of hospital stakeholders across WA on factors that impact patient outcomes and to describe the development of the causal model used in the final phase of the study.

## METHODS

### Study Design

We conducted a qualitative study using semi-structured interviews and thematic analysis to understand hospital stakeholder perspectives and their experiences of factors that affect acute care inpatient outcomes. As part of our causal modeling, qualitative research methods were chosen to explore relationships between variables, including context, mechanisms, and outcomes.^4^ This exploratory approach also acknowledges the significance of subjectivity in the data and allows for inductive inquiry.^5^ The findings from this study served as a foundation for developing a comprehensive model we called the ‘Washington Acute Care hospital Characteristics and patient Outcomes model’ (WACCHO), which considers community, hospital, and patient characteristics that interact with staffing to affect patient outcomes. This study was granted exempt status by the University of WA and the WA Institutional Review Boards (STUDY00013975).

### Participant Recruitment

Participants were purposively sampled through announcements to state-wide hospital email listservs. Hospital executives and administrators were also directly emailed to increase participation and ensure participants represented diverse hospital types and geographic settings. Site contacts were asked to invite any hospital representative who could provide perceptions of staffing’s impact on patient outcomes. Participants were unknown to the research team prior to the interviews.

### Data Collection

The preliminary model from study phase 2 was used to develop a semi-structured interview guide, which was tailored for each site with a few hospital-specific data points and shared with participants prior to the interview. Open-ended questions explored factors, mechanisms, contextual elements, and additional variables that could potentially influence patient outcomes in 4 main categories: hospital characteristics and external factors, patient characteristics, staffing characteristics, and patient outcomes. Interviewers also presented facility-specific data, asked participants for their perceptions of accuracy, and discussed the basic analysis plan for the quantitative portion of the study. A list of interview questions and prompts is provided in an additional file (Additional File 1). Interviews were conducted between January and June 2022 via video conferencing by 2–5 members of the research team (SI, AF, NBS, NH, AP). Each participant was interviewed once, and all interviews were audio recorded and transcribed for analysis. The research team introduced themselves and explained the purpose of the study. Upon obtaining oral consent from participants, 2 members of the research team (SI, NH) took detailed field notes. Transcripts were uploaded to ATLAS.ti (version 9).

### Analysis

Both deductive and inductive methods were used in the thematic analysis of data.^5^ An initial codebook was created based on our initial model and interview notes, and emergent codes were added inductively during analysis.^5^ Five team members (NH, JZ, ED, KN, KB) contributed to coding. They met weekly to review codes, ensure a uniform interpretation and application of the coding framework, and address any discrepancies. A portion of each transcript was coded by at least 2 researchers to ensure consistency. Once coding was completed, codes were iteratively organized into main themes and subthemes to capture the range of narratives.^5^ Saturation was determined when no new themes were identified in final interviews. We followed the consolidated criteria for reporting qualitative research guidelines (COREQ) to ensure comprehensive reporting.^6^

### Limitations

This study had several notable limitations. First, the timing of interviews during the COVID-19 pandemic made it challenging for hospital representatives to participate. Frontline staff were often unavailable, and leaders were frequently supporting patient care activities during surges in admissions. This resulted in less robust representation from some healthcare team members and more prominent representation of executive and administrator perspectives, which may have focused the discussion on nurse staffing structures rather than perceptions of patient needs. Second, as the study focused on experiences before the pandemic, participants were asked to remember past perceptions, which challenged their focus and could have led to limited recall bias. Third, our team’s innovative approach to expanding staffing models may have challenged participants to think beyond their current perceptions and narrowed their feedback. Finally, as our causal model was iteratively developed throughout the interview period, interview questions were not static and discussion may have focused on elements that stakeholders felt more strongly about, influencing the quantity of participant feedback on specific elements of the model.

## RESULTS

### Participants

A total of 20 interviews were conducted with 38 participants from 19 hospitals in 8 out of 9 regions across WA. Participants worked at 3 main types of hospitals: acute care (23/38), critical access (11/38), and sole community hospitals (4/38). While the definitions of hospital types may vary in some literature, the Centers for Medicare and Medicaid Services (CMS) officially designates critical access and sole community hospitals as specific types of acute care hospitals, where critical access hospitals are typically smaller and located in rural settings.^7,8^ Participants included a broad range of executives and administrators (23/38), directors and managers (10/38), and interprofessional care team members (5/38) from all 3 hospital types. Mean interview length was 61 minutes.

### Causal Model

**Figure 1:**
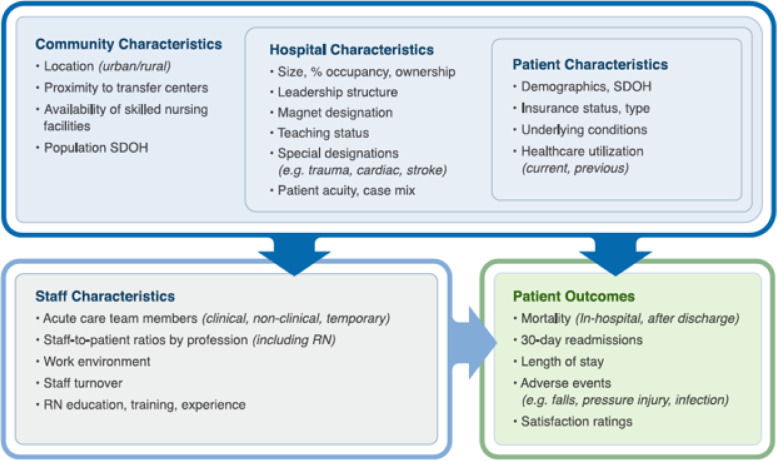
Final explanatory model of factors impacting patient outcomes, the Washington Acute Care hospital Characteristics and patient Outcomes model (WACCHO)

The final model shows the primary factors and drivers impacting patient outcomes as agreed upon by participants (Fig. 1), which includes reorganized categories from the initial model to better represent findings. Hospital characteristics and external factors were divided into two distinct categories, with external factors renamed as community characteristics. The reorganized model nested patient characteristics into hospital characteristics, and in turn these were nested within community characteristics to highlight the relationships between the three categories. The new community characteristics category, inclusive of hospital characteristics and patient characteristics, impacts staffing characteristics and patient outcomes, and staffing and patient outcomes are directly connected. Findings are presented here by model category and Table 1 summarizes themes and their frequency.

### Community Characteristics

Community characteristics were defined as the setting in which the hospital exists. This setting includes factors outside of the hospital’s control, such as sociopolitical, geographical, and economic factors, and availability of other healthcare resources.

#### Location and community resources.

Participants often described the difficulty of discharging patients to the appropriate level of care, meaning the resources and facilities needed to meet patient needs and including higher acuity care such as transfer to a referral hospital, or subacute care such as discharge to a skilled nursing facility. When resources for the appropriate level of care are limited, the hospital must keep patients in acute care beds, limiting available resources for other patients. These challenges were more pronounced in rural settings with fewer community resources.

Community characteristics also impacted staffing. Both rural and urban participants described how location and community resources like affordable housing, public transportation, and commute times made it difficult to recruit and retain hospital staff. Additionally, participants in rural locations noted the difficulty in recruiting staff when they are not close to or connected with teaching institutions producing new graduates.

#### Population.

Participants discussed their facilities’ unique challenges due to the populations they served and health disparities present within the community. Participants listed characteristics such as homelessness, homes without basic utilities (e.g., running water or electricity), health insurance in the community, and transient seasonal populations including migrant workers and summer tourists.

### Hospital Characteristics

Hospital characteristics were defined as the structural and functional qualities of acute care hospitals that influenced the services they offered and the complexity of patients they served.

#### Hospital type and access to resources.

Participants stated that their hospital type, specifically size and connection to larger health systems, influenced their access to resources. Various stakeholders described limited budgets and reduced access to equipment secondary to supply chain constraints. Critical access hospitals identified their smaller size and limited resource pool as reasons they must be more particular with capital investments that would enable them to care for more complex patients while simultaneously having the obligation to provide specialty services that are not otherwise available in their communities. Participants from critical access hospitals also described lack of access to the relationships and shared knowledge within larger health systems, which they felt negatively impacted their efficiency in rolling out new policies and processes.

#### Hospital leadership structure and culture as foundational to quality.

Participants considered staffing and leadership culture as a product of organizational priorities that influenced staff satisfaction and quality outcomes. Participants identified that characteristics including union status, staffing strategies, budget, and patient care equipment influenced staffing and leadership culture. Stakeholders cited efforts by the organization to ensure adequate equipment and supplies as important to providing quality patient care. As one acute care hospital administrator described, "*Something as simple as an overbed table… When we talked about this at incident command…the answer was no. And then, thank God, our CEO is also a nurse and she’s like no, this is basic to taking care of patients and keeping them from falling*.”

Participants noted organizational features that emphasized safety culture, with elements like care quality and improved organizational processes. Multiple participants referenced standardized protocols as a safety tool that contributed to improved patient care. Participants also felt an organizational focus on safety and transparency improved staff satisfaction and quality of care. Comments referenced the importance of continuous quality improvement and a focus on process improvement instead of individual errors.

#### Influence of organizational culture on staff retention.

Participants agreed that the culture of an organization influenced work environment and staff retention. They described approaches to support and engage with staff which promoted a positive organizational culture. One approach included providing staff with incentives and benefits such as increased pay, bonuses, parking passes, flexible shifts and scheduling. Other examples included programs which covered the cost of nursing education in exchange for commitment to work in a given facility for a period of time. Participants also presented upstream approaches which improved the work environment, such as involving workers in organizational decision making and appropriate staffing of the interprofessional team. Overall, as one interprofessional care team member stated, “*if you’re not given the tools to do your job well, anybody with any empathy is going to go find something else to do*.”

#### Units vary across and within hospitals.

When discussing data metrics, participants often discussed the difficulty in making comparisons of the same unit between different hospitals and comparisons of units within the same hospital. They expressed confusion with how acuity is defined, especially when comparing patient care across different facilities. Participants felt it was too difficult to use case mix index, a metric used to identify the diversity and severity of patients cared for at specific hospitals, to compare outcomes between units within a hospital or across healthcare systems. Participants did not think case mix encompasses all the variables that should be considered when evaluating the complexities of the patient and the care infrastructure.

### Patient Characteristics

Patient characteristics are defined as individual demographic, social, and health characteristics of patients admitted to the hospital that may impact the level of care needed.

#### Underlying health conditions impact the intensity of care.

Participants used the term ‘care intensity’ to describe how patient care needs impacted work demands on staff, with agreement that the care intensity is not always directly tied to the patient’s admitting diagnoses or assigned acuity. Participants reported this disparity between acuity and care intensity as a challenge to accurately predict staffing needs. They noted that specific health conditions with higher care intensity included aggressive behavior, traumatic brain injury, obesity, substance use, and dementia. Participants described different strategies to account for care intensity variations, such as having a centralized staffing office or a predefined team who coordinated activities to accommodate rapid and fluctuating changes in staffing needs. In addition to increased care intensity and inpatient staffing demands, patients with certain underlying conditions were difficult to discharge due to the availability of appropriate care in the community or required social support, such as individuals needing guardian assignment.

#### Social history and economic characteristics impact on health status.

In addition to descriptors of the population served in the community characteristics section, the demographics, social determinants of health (SDOH), and insurance status of patients influenced their care needs. Factors such as access to routine care, prior healthcare utilization, and comorbidities impacted care intensity and needed resources.

### Staffing Characteristics

Staffing characteristics are defined as acute care team members, their roles, and aspects of staffing which influence how facilities provide staff and deliver patient care.

#### Interprofessional acute care team composition and the central role of nurses.

When considering the relationship between staffing and patient outcomes, participants discussed team members who contribute to the care team and work in tandem to provide patient care. Participants mentioned roles in multiple professions including physicians, advanced practice providers, RNs, certified nursing assistants, occupational and physical therapists (PT), pharmacists, social workers, dietary aides, environmental service workers, billing/coding staff, students, and others. Care team members were generally categorized as either clinical, non-clinical, or temporary roles. There was a lack of agreement around the types and quantities of roles included in the acute care team. However, participants discussed state mandated annual RN staffing plans and nurse-to-patient ratios, highlighting the central role and value placed on RNs in acute patient care and interprofessional care teams.

#### Influence of staffing type on work environment.

Participants emphasized the importance of differentiating between temporary (e.g., contract, agency, or travel) and permanent RNs when examining how staffing impacted patient outcomes. Stakeholders expressed that temporary workers may be less familiar with facility policies and may not have the same unit-specific training as permanent staff. Additionally, facilities with a larger proportion of rotating temporary workers may not have an established culture of communication and support, which diminishes the quality of the work environment and negatively influences patient outcomes.

#### RN absorption of non-nursing duties resulting in the dilution of nursing care roles.

Although facilities submit annual nurse staffing matrices, participants frequently spoke to the need to deviate from planned models, highlighting variation in direct and indirect patient support staff which make nurse-to-patient ratios in one setting incomparable to the same workload in another setting. Participants also presented instances when facilities had difficulty filling staffing roles, so RNs absorbed responsibilities, diluting the scope of nursing practice. For example, one sole community hospital administrator stated, “*If you're short PT assistants or PT aids, that falls back on the RN and the nursing assistant. If you donť have case management or social work, that also falls on the RN. Everything falls on the RN, if. .. the rest of the team is missing*.”

#### Education, training, and experience.

Discussions around education, training, and experience centralized around nursing staff and largely focused on the nuances of the term ‘experience’. Participants agreed that RN experience was complex and difficult to capture, quantify and standardize. Various metrics for measuring experience were presented and considered, such as years of RN or inpatient experience and unit tenure. Participants also quantified RN experience with standards such as a novice to expert or years since licensure. Degrees, licenses, and certifications were discussed as components of education, with several participants stating that nurse training was not well documented except in human resource records. Participants noted that overall training and experience on the unit influenced the ability to staff appropriately for patient acuity and diagnosis. When units had higher numbers of staff with more training and experience, the unit could manage more complex patients, yet in many locations, the limited number of experienced staff made patient assignments difficult.

### Patient Outcomes

Patient outcomes describe metrics pertaining to characteristics of a patienťs stay at a hospital and the time immediately following discharge, which are a collection of quality and safety metrics tracked by the hospital and the state.

#### Impact of staffing on patient outcomes.

When asked about patient outcomes, participants described some measures as more sensitive to staffing than others. Participants characterized staffing-susceptible outcomes as being dependent on care team composition and staffing type rather than number of staff or staff-to-patient ratios. Participants specifically mentioned falls and pressure ulcers as staffing-sensitive outcomes, with one hospital administrator noting that, “*one of the things…making a significant impact on patient outcomes or patient satisfaction and staffing is the number of non-hospital nurses that we have here. So, we have 72 travelers, and we have FEMA [Federal Emergency Management Agency] staff, and so our fall rates increased, our HAPIs [Hospital-Acquired Pressure Injuries] have increased, complaints have increased*.”

#### Cumulative impact of model themes on patient outcomes.

Participants discussed how some patient outcomes like length of stay or readmission are significantly impacted by model categories outside of staffing. One example was length-of-stay, defined as the number of days a patient is cared for in an acute care facility. Several participants described the influence of community and patient characteristics on length-of-stay related to needs for social support or availability of a skilled nursing facility bed. One critical access hospital administrator stated, “*it happens, where we cannot get a patient out. We don’t have a receiving hospital or we don’t have EMS. .. that’s the challenge of being. .. rural*.”

## DISCUSSION

This study produced critical findings on factors influencing staffing and patient outcomes in the acute care setting. While some findings reinforce existing knowledge such as the importance of adequate RN staffing, others identify gaps in both knowledge and theory. While the discussion highlights the gaps in each of the categories in our causal model, the gaps are often interconnected and responsive to dynamic changes in other model components. For example, changes in hospital leadership may impact both hospital and staffing characteristics in ways that subsequently change patient outcomes. We also note a need to expand future work to examine the impacts of the pandemic, as this work focused on pre-pandemic experiences.

### Community Characteristics

Participants practiced in a wide array of settings and consistently brought forward the need to account for different contexts when considering staffing and related policy. Findings suggest that a ‘one size fits all’ approach to staffing is undesirable, instead emphasizing the need for individual organizations to account for their communities and settings when establishing staffing standards. This viewpoint is consistent with implementation science theories such as the Consolidated Framework for Implementation Research,^9^ which emphasizes the inclusion of contextual factors when planning, developing, implementing, and evaluating a practice or policy change. Accounting for community contexts allows organizations to attend to the populations they serve and the resources available in their settings. For example, communities with lower demand for inpatient beds and more limited access to skilled nursing facilities may need the flexibility to provide a lower level of care (e.g., a higher patient to nurse ratio) when a patient ready for skilled nursing is still physically present in the hospital.

### Hospital Characteristics

While organizational culture has been linked with workforce outcomes such as RN turnover and retention,^10^ participants indicated that elements of culture are also vital to conversations about staffing and patient outcomes. An organizational emphasis on safety and just culture provides opportunities for workers to provide input on staffing needs and challenges. Within just culture, transparency and psychological safety work bidirectionally to ensure that staff can bring forward concerns without penalty and that management and administration share information on their own challenges and progress related to staffing.^11^

Several proven strategies for approaching this type of culture are Magnet^®^ designation, which emphasizes the involvement of RNs in hospital administration, policy, and practice,^12^ and American Association of Critical Care Nurses’ Healthy Work Environment, which identifies 6 critical elements to a just unit culture.^13^ Accounting for features of organizational culture using an established framework such as these would help provide additional information and clarity into organizational practices around staffing, which may be an important predictor or mediator of the relationship between staffing and patient outcomes.

Hospital environment and culture impact patient care in other ways. For example, one participant’s recollection of a discussion about bedside tables shows how a leader with bedside experience recognized the importance of a piece of equipment in promoting patient safety. In addition to these administrative types of decisions, structural and logistical features of hospitals impact staffing and workload. For example, when patient care supplies were not readily available, nurses or other direct care staff had to leave the unit to retrieve them, taking time and focus away from patient care.

### Patient Characteristics

Patients with different types of acute care issues have various levels of need, often represented in terms of patient acuity or some measure of nursing hours invested in care.^3^ In our study, despite consensus across participants that the unique care needs of individual patients are not routinely captured in acuity measures or admitting diagnoses, there was no agreement on a standardized way these needs could be measured or reported. High care needs, particularly those stemming from the intersection of behavioral, mental, and physical health status, require additional work from the care team. Participants indicated that these situations disrupt the uniťs workflow and change staffing needs, even when no additional staff are available. While care quality measures aim to increase inpatient assessment of SDOH, this evaluation may indicate a need for more resources than staff have available to address issues. Overall, a more nuanced understanding of patient needs as they affect the unit workload is necessary when evaluating staffing practice and policies.

### Staffing Characteristics

Staffing has been a topic of interest in health services literature for decades, with most data focused on RN staffing levels.^1^ One of the main issues identified in our scoping review and reiterated by participants in this study was the lack of interprofessional team member inclusion in staffing plans.^3^ Participants brought forward concerns about what work the RN is doing when other staff are missing and how doing that work impacted their availability to perform needed nursing tasks. Diluting RN time with non-nursing tasks means that RNs are not working at the top of their scope of practice, which leads to dissatisfaction and connects to burnout and turnover.^14^ Similarly, when there are not enough RNs with the training and experience to care for certain types of patients, patient outcomes may suffer.^15^

### Patient Outcomes

When assessing patient outcomes in health services research, data are often sourced from statewide administrative bodies and include a range of quality metrics such as falls, skin breakdown, length of stay, mortality, and patient satisfaction. While measures like falls and skin breakdown are often labeled as “nursing sensitive”, participants indicated that nurses are not the only staff members whose presence or tasks may impact those outcomes. For example, if typical resources such as PT/aides are unavailable to ambulate a patient, the RN may not be able to add that task to their workload, leading to skin breakdown. In this case, the ‘nurse-sensitive’ indicator may not tell the whole story about staffing.

Other patient outcomes like length of stay or readmission may be more indicative of community resources. For example, the availability or staffing levels of residential facilities that care for patients with sequelae of brain injury may mean that patients linger in the acute care setting or get sent back to the emergency room if facility staff are unable to handle symptoms. These types of influences are rarely accounted for in studies which focus on direct measures of nursing staffing and patient outcomes in acute care.

Patient outcomes also vary when underlying conditions or characteristics, including SDOH, impact overall health and the ability to provide needed services in the acute care setting. WA state now requires hospitals to report certain data on SDOH to the Department of Health,^2^ which will improve the ability to account for these characteristics in future analyses of patient outcomes and provide more conclusive evidence related to health equity in different patients and communities.

### Policy Implications

Throughout the study, stakeholders noted the difficulty of applying a blanket policy to individual organizations. Instead, they identified a need for a more nuanced understanding of individual hospitals when setting staffing standards. When aiming to ensure safe staffing levels at the local, state, or national level, policy needs to reflect more than just the numbers of a specific type of staff at the bedside, instead drawing on a more comprehensive understanding of the communities, settings, and patients served at different facilities. This process may require more robust data collection and commitment to ensuring an adequate supply of workers.

## CONCLUSION

Altogether, this study enhanced the initial findings of our scoping review by providing insight from healthcare stakeholders in various types of acute care hospitals across the state. Findings highlight the complexity and interrelatedness of the categories in the causal model, while drawing attention to critical gaps that must be addressed to better understand how communities, organizations, patients, and staffing all impact patient outcomes. Our study highlights the need to ensure that RN-centered care teams include appropriate interprofessional staffing to meet the needs of patients, and that access to community resources is critical both for ensuring that patients receive efficient continuity of care throughout their recovery and that acute care beds and staff are appropriately used. Future research should expand on this study to better understand lessons learned from the COVID-19 pandemic, with specific attention to staffing changes and interprofessional team composition that can direct future work to improve patient outcomes. Ensuring optimal staffing of interprofessional teams also has the potential to decrease burnout, leading to improved outcomes for interprofessional acute care staff and improved retention of this vital workforce.

## Figures and Tables

**Table 1:** Summary of themes by model category and frequency

	Theme	Frequency (# hospitals reporting)
**Community Characteristics**	Challenges due to location: patients, staffing, and community resources	13
	Characteristics of the population	15
**Hospital Characteristics**	Hospital type influences access to resources	19
	Hospital leadership structure and culture as foundational to quality	19
	Units vary across and within hospitals	16
**Patient Characteristics**	Underlying health conditions impact the intensity of care	16
	Social history and economic characteristics impact on health status	15
**Staffing Characteristics**	Interprofessional acute care team composition	17
	Interprofessional acute care team centralizes around nursing	8
	Nurse absorption of non-nursing duties resulting in the dilution of nursing care roles	18
	Influence of organizational culture on work environment	15
	Education, training, experience	18
**Patient Outcomes**	Impact of staffing on patient outcomes	12
	Cumulative impact of model themes on patient outcomes	11

## Abbreviations

RN: Registered nurse
WACCHO: Washington Acute Care hospital Characteristics and patient Outcomes model
CMS: Centers for Medicare and Medicaid Services
CEO: Chief executive officer
PT: Physical therapist
FEMA: Federal Emergency Management Agency
HAPI: Hospital-acquired pressure injury
SDOH: Social determinants of health
